# Robotic ultrasound scanning platform with autonomous control, multimodal human–machine interface and real-time image analysis

**DOI:** 10.1007/s11548-026-03763-4

**Published:** 2026-07-30

**Authors:** Rafael Benito, Laura Pérez Sánchez, Amaia Iribar-Zabala, Marco Ojer, Marcos Garro, Verónica de Ramos, Jon Ortega, Álvaro Bertelsen, Xiao Lin, Ignacio Sánchez-Varo, Lucía Salazar, Juan Alberto Sánchez-Margallo, Mikael Brudfors, Nadim Daher, Karen López-Linares, Davide Scorza, Miguel Ángel González Ballester

**Affiliations:** 1 Vicomtech Foundation, Basque Research and Technology Alliance (BRTA), San Sebastian, Spain; 2 BCN Medtech, Universitat Pompeu Fabra, Barcelona, Spain; 3 Departamento de Bioingeniería, Universidad Carlos III de Madrid, Leganés, Spain; 4 eHealth group, Biogipuzkoa Health Research Institute, San Sebastian, Spain; 5 Bioengineering and Health Technologies Unit, Jesús Usón Minimally Invasive Surgery Centre, Cáceres, Spain; 6 NVIDIA, Santa Clara, CA USA; 7 Cyber Surgery, San Sebastian, Spain; 8 ICREA, Barcelona, Spain

**Keywords:** Surgical robotics, Autonomous robotics, Ultrasound scanning, Surgical navigation

## Abstract

**Purpose:**

Autonomous robots can streamline repetitive and time-consuming surgical tasks like ultrasound scanning. AI can provide the necessary intelligence, but for clinical acceptance, systems must move predictably, offer intuitive interaction, and maintain low latency on medical-grade hardware.

**Methods:**

We present an integral robotic platform for autonomous ultrasound scanning. The platform allows to perform scanning on either manual or autonomous mode. The autonomous control combines AI-driven ultrasound target segmentation with geometric motion algorithms designed to focus the probe on the segmented target. We optimized the image processing models for specialized hardware to ensure real-time performance with constrained resources. Users can control the platform via natural language voice commands processed by a Large Language Model (LLM) and visualize the procedure through a synchronized 3D Digital Twin and an augmented reality (AR) environment.

**Results:**

We tested the platform in hepatic tumor localization in a synthetic phantom. Autonomous tumor localization showed a success rate of 84% with an average execution time of 5.75 s.

**Conclusions:**

Our system successfully automates ultrasound tasks, allowing the user to control the process through an intuitive, multimodal interface. By pairing optimized AI with specialized hardware, we achieved low-latency, real-time adaptability with minimal hardware resources. In the future we plan to adapt this platform for more complex neurosurgical and urological procedures.

Introduction

Automating simple surgical tasks reduces surgeon workload, allowing focus on complex reasoning and dexterity. However, commercial surgical robots lack significant autonomy. The Da Vinci [1], for instance, requires continuous human control. While some systems can automate tasks in hard tissue (bone) surgery [2–4], soft tissue remains a challenge due to its unpredictable nature.

Academic researchers develop various autonomous systems, primarily using reinforcement and imitation learning [5]. More recently, vision–language–action (VLA) models have increased task versatility and complexity [6, 7]. However, these methods suffer from two shortcomings. First, they require massive datasets, yet real-world surgical data remains scarce. Second, “black box” AI models complicate regulation; their complex decision-making processes lack the transparency and predictability crucial for surgery. Besides the involved methodology and algorithms, researchers rarely address another key point: real-world hardware limitations. Modern operating room workflows demand specifically tailored hardware, which most research-tier solutions fail to meet because developers built them in laboratory environments

In this work we focus on automating ultrasound scanning; a task that, although not too complex, demands a lot of time and effort for human surgeons. In autonomous robotic ultrasound, a common control strategy involves visual servoing, where real-time ultrasound video is used to guide the robot’s movement and, for instance, make it reach a certain target visible in the image. Most recent approaches use AI for both image processing and sophisticated robotic control [8–11]. More traditional approaches use heuristically designed geometric algorithms for robot movement [12, 13]. Even if the capabilities are somewhat limited, these traditional methods are simpler and more predictable.

In this work, we aim to develop a platform that merges state-of-the-art AI-based ultrasound analysis with a more traditional control strategy based on deterministic geometric algorithms, deploying everything on hardware that can be seamlessly integrated in real-world surgical workflows. The platform works based on three pillars: motion planning based on the deterministic algorithms, real-time AI image analysis for ultrasound target recognition and an intuitive human–machine interface featuring natural voice commands, a 3D Digital Twin and AR visualization. Rather than advancing on the development of one specific technology, the main contribution of this paper is on the merging of many state-of-the-art technologies into a unified platform that provides predictable behavior with an intuitive user interface and considers real-world hardware constraints.

We validated the platform for the scenario of hepatic tumor ablation by localizing and tracking liver tumors in a synthetic phantom.

## Methods

We describe our platform’s composition in three subsections. SectionWorkflow details the platform’s overall workflow and operation. Section System architecture outlines the system architecture and its components, while Section Human–machine interface explains the experimental setup we developed to test the platform.

### Workflow

**Fig. 1:**
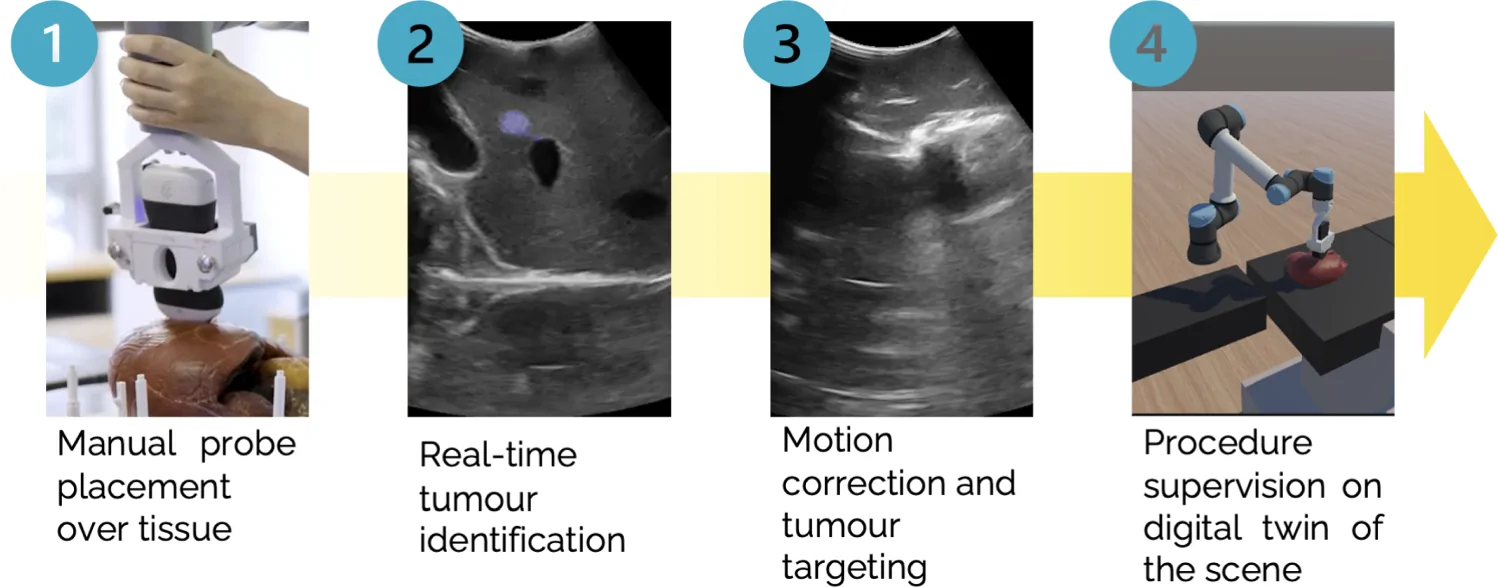
Workflow in autonomous ultrasound scanning mode. User provides manual initialization, and then, the robot is capable of autonomously localizing and tracking the tumor

Our platform centers on a robotic arm operating an ultrasound probe. In manual mode, the arm functions as a collaborative robot (cobot). In autonomous mode (see Fig. 1), the robot maintains constant vertical force against the phantom to ensure high-quality imaging. As the arm sweeps the phantom’s surface, it streams ultrasound video to an AI model that segments tumors and calculates their coordinates in real-time. The results are fed to the robotic control module, which centers the probe over the detected tumor.

Users can interact with the system through three main interfaces. First, they switch operating modes (e.g., manual, automatic, or emergency stop) using voice commands processed by a Large Language Model (LLM). Second, a Digital Twin renders the surgical scene in real-time, allowing users to monitor the robot-phantom interaction. Finally, an augmented reality (AR) interface streams the ultrasound video and tumor segmentations directly to a specialized headset.

### System architecture

**Fig. 2:**
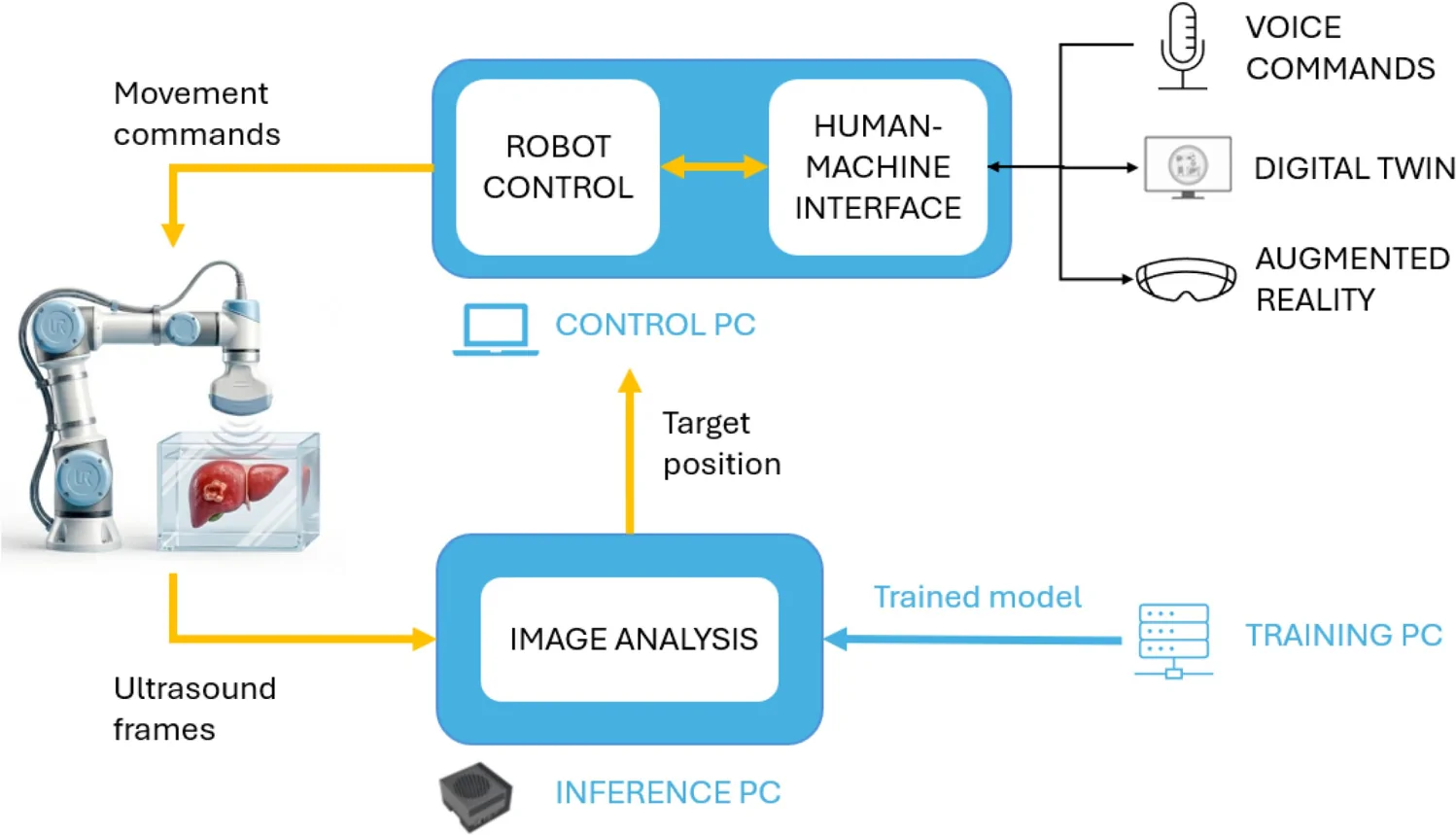
Overview of the platform. It works based on three computers (in blue) that host and support the three modules of the platform (white boxes)

Figure 2 illustrates the platform’s architecture. We distributed the system across three computers, inspired by the “Three Computer Solution” for autonomous robotics [14]:

- **Control PC:** Executes the robot control logic by integrating ultrasound feedback and user commands. It also hosts the human–machine interface (HMI) for real-time interaction and visualization. We used an Alienware 17 R4 laptop with Windows OS, but any common desk PC or laptop would suffice, since there are no extraordinary hardware requirements.
- **Inference PC:** It holds the image analysis AI model that receives the ultrasound video from the probe and segments the tumor(s) in real-time, sending their position to the robotic control PC. To ensure that the system could work with low latencies in restricted environments like the operating room, we used as inference PC a Jetson AGX Orin 64GB [15], which is optimized to be spatially efficient, operate with minimal power-to-performance ratio and minimize thermal and acoustic impact.
- **Training PC:** Before deploying on Jetson, the image analysis model was trained on a dedicated server with two GeForce RTX 2080 and 3060 GPUs. While the training PC does not directly operate the platform, it continuously retrains and improves the underlying AI models using newly acquired data.

Inspired by the modular architecture of the MIGHTY Platform [16], the system organizes functionality into three interconnected modules: human–machine interface, robotic control module and image analysis module. We used the industry-standard OPC-UA protocol [17] to manage communication between modules. Below, we describe the details on each of the three modules.

#### Human–machine interface

Our multimodal interface enables robot interaction through natural voice commands and real-time visualization via a 3D Digital Twin or an augmented reality (AR) headset.

**Fig. 3:**
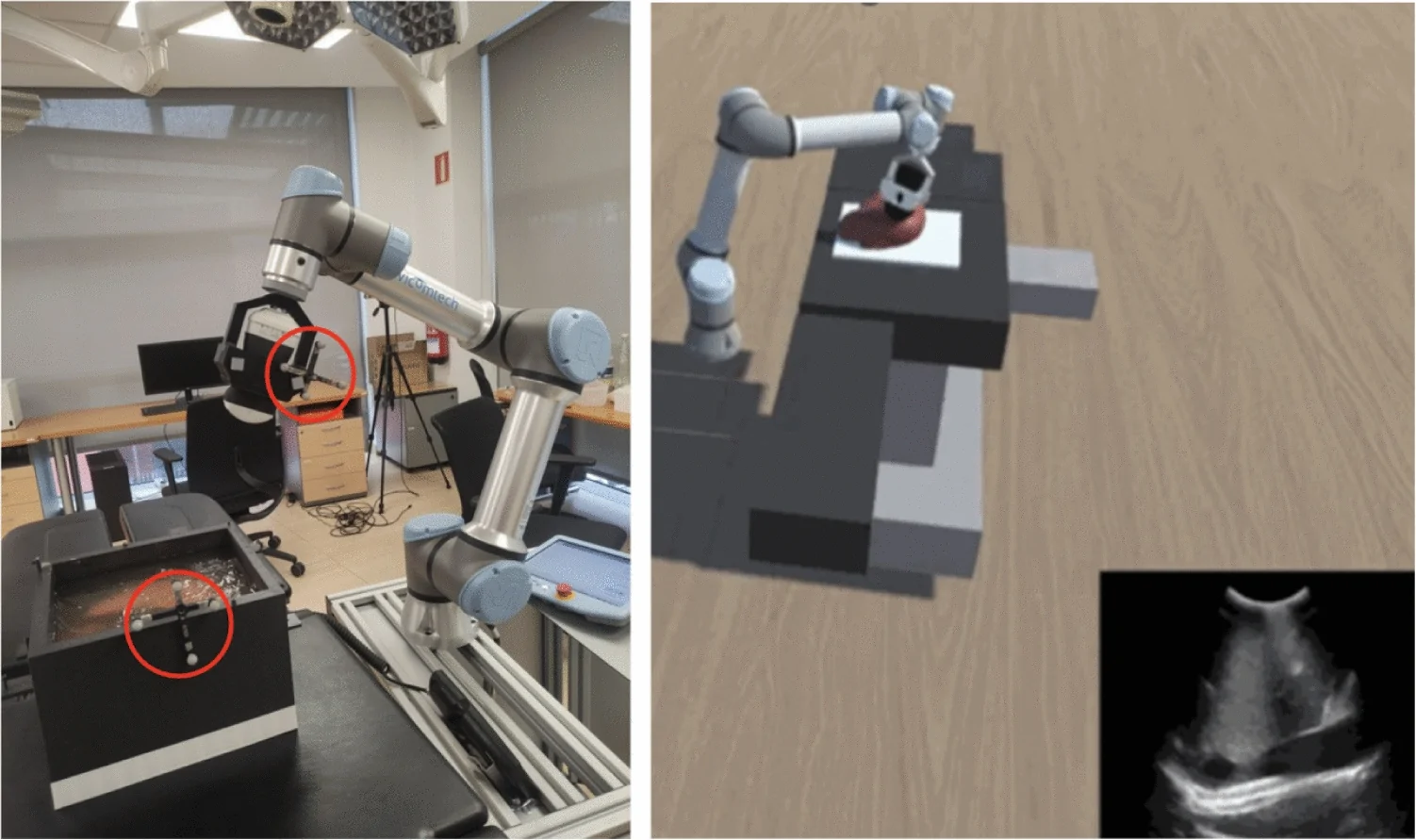
Left: Real surgical scene, with the robot and the phantom. The optical markers used for calibration can be seen circled in red. Right: Virtual representation of the scene in the Digital Twin. The ultrasound image is displayed in the bottom right corner

**Voice Commands.** We use natural language to switch between the following operating modes:

- *Approach to Workspace*: Positions the robot above the phantom.
- *Retract from Workspace*: Moves the probe to a safe, retracted position.
- *Enter Manual Mode*: Activates cobot functionality for manual handling.
- *Localize Tumor* (Automatic Mode): Triggers autonomous scanning.
- *Stop*: Immediately halts all movement and locks the robot’s position.

We transcribe speech using OpenAI Whisper [18] and process the text through a Google Gemini [19] agent to identify the intended command. For example, if a user says, “OK, let me handle the probe,” the agent triggers *Manual Mode*. To prevent accidental activation from background noise, we use the hot-word “Arturito”; the Gemini agent only reacts to sentences preceded by this trigger. A synthetic voice provides immediate feedback once the system receives and executes a voice command.

**Digital Twin.** We developed a virtual representation of the surgical scene (Fig. 3) that mirrors the robot-phantom interaction. The 3D model of the phantom or patient can be obtained from preoperative medical image segmentation. To track the phantom’s relative location with respect to the bed and the robot, we attach spherical retroreflective markers to both the robot flange and the phantom, and monitor them by an OptiTrack V120:Duo (Natural Point, Inc., Corvallis, OR, USA) tracker [20]. Because we mechanically fix the robot to the bed, the bed’s position remains constant relative to the robot. Similarly, an ad hoc CAD scaffold attaches the ultrasound probe to the robot, keeping its pose fixed and known relative to the robot flange.

Inspired by previous methods used in endoscopy [21, 22], we developed a calibration program that allows determining the position of the phantom with respect to the global frame and the robot base. Knowing a pose of the ultrasound probe (U) with respect to the robot base (R) (${}^{R}T_{U}$), the same pose of the probe with respect to the optical tracker (W) (${}^{W}T_{U}$) and the pose of the phantom (P) with respect to the tracker (${}^{W}T_{P}$), we can compute the pose of P with respect to the robot using equation 1.

1 $$\begin{aligned} {}^{R}T_{P} = {}^{R}T_{U} \cdot ({}^{W}T_{U})^{-1} \cdot {}^{W}T_{P} \end{aligned}$$

We compute this transformation across four distinct robot poses to ensure accuracy and robustness.

**Fig. 4:**
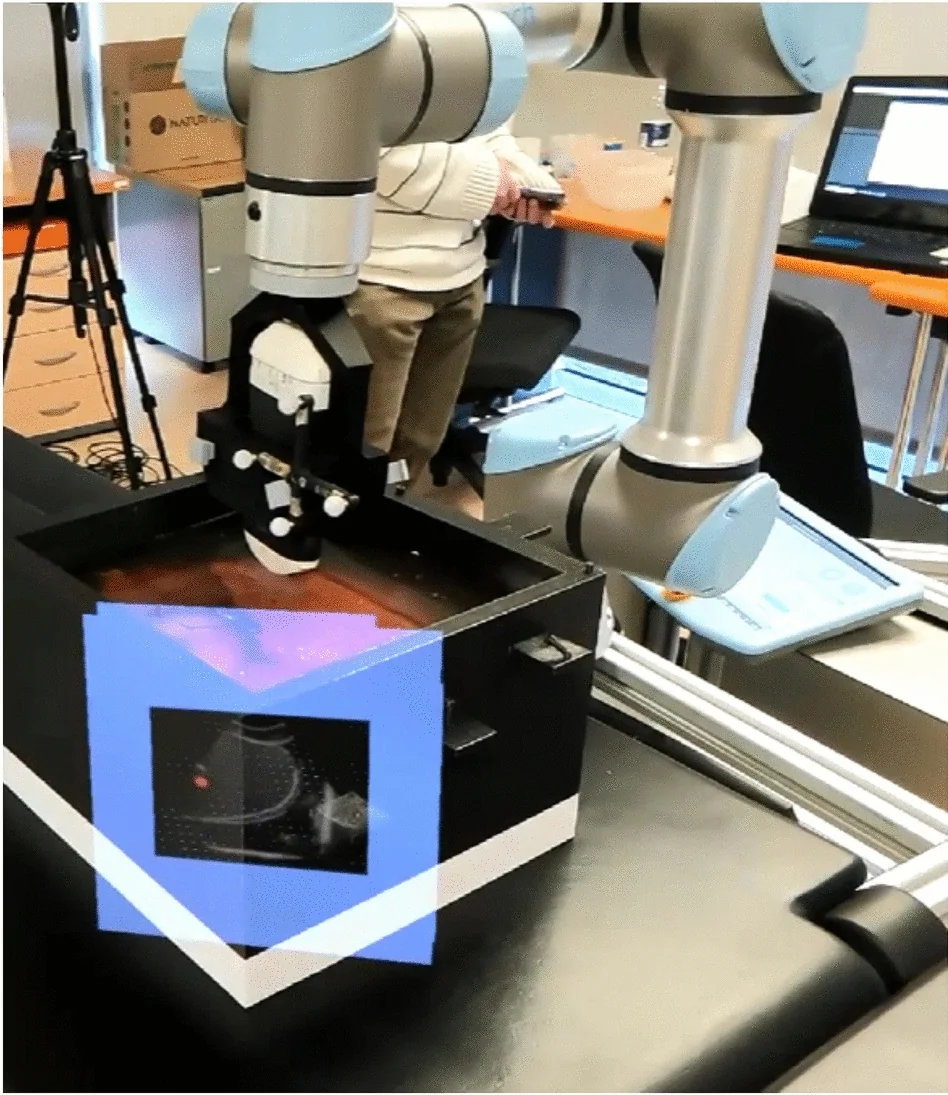
AR visualization, from the user’s perspective. The window with the ultrasound image can be found over the black frame of the phantom

**AR interface.** The AR interface was developed using the Unity Engine 2022.3 LTS [23] and the Mixed Reality Toolkit (MRTK) [24] with the Magic Leap 2 [25] headset. A dedicated server streams real-time ultrasound frames and tumor segmentations to the headset via the OpenIGTLink protocol. Users view the segmented ultrasound video in a floating viewport, which they can scale, rotate, or reposition using hand gestures to suit their spatial constraints during surgery (Fig. 4).

#### Robotic control module

We use a Universal Robots UR5e collaborative manipulator to handle the ultrasound probe. The robot receives high-level commands from the HMI, and each command triggers a specific control strategy:

- **Approach to / Retract from Workspace**: The robot moves to predefined coordinates using Cartesian position control. Here, target end-effector poses are transformed via inverse kinematics into joint-space trajectories, which are executed using the robot’s native low-level PID position controllers.
- **Enter Manual Mode**: The robot enters hand-guiding mode, allowing the operator to guide the end-effector manually.
- **Localize Tumor**: The robot executes an autonomous search using a hybrid control scheme. Following the force magnitude standards reported in the literature [26], it applies a constant force of 10 N along the vertical (Z) axis while maintaining position control in the horizontal plane and rotational degrees of freedom. As the robot sweeps the search volume, it monitors real-time tumor coordinates from the image analysis module. If no tumor is detected, the robot executes a back-and-forth motion perpendicular to the ultrasound image plane, constrained within predefined limits, to explore the search volume. If the image analysis module detects a tumor, the robot adjusts its position to center the target within the ultrasound field of view. When moving to the tumor, rather than sending the robot to a fixed 3D pose, the movement is commanded in small incremental steps and controlled in closed-loop, evaluating the reduction of the voxel-wise distance from the tumor center to the vertical center of the image field of view.
- **Stop**: The robot halts immediately and holds its current pose using position control. The stop signal can be sent either through voice commands or through a safety button that is always available.

#### Image analysis module

**Fig. 5:**
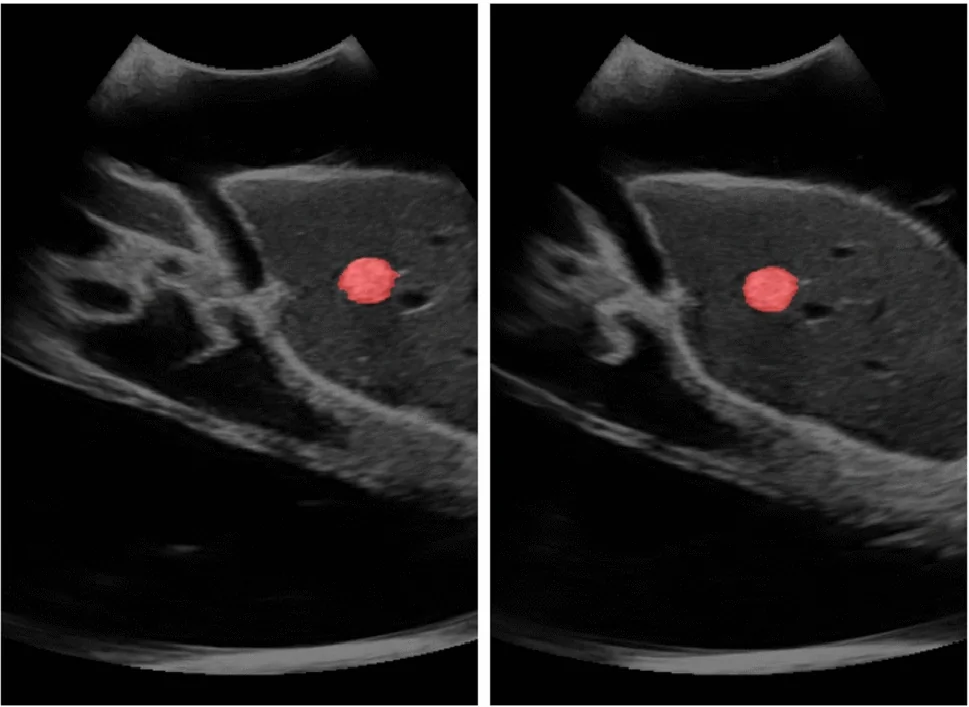
Segmented ultrasound images displaying the gradual tumor centering procedure. The segmented tumor appears colored in red

The image analysis module processes ultrasound video frames to generate binary tumor masks. It also calculates the 2D distance between the tumor’s centroid and the center of the ultrasound field of view and then transmits these coordinates to the robotic control module, so it can center the probe on the tumor (Fig. 5).

We implemented a Flexible U-Net architecture from the MONAI Model Zoo [27], training it on 1,957 manually annotated ultrasound frames and validating it on 419. All training and validation images were captured over the validation phantom (see section Experimental setup). During inference, the network processes 640×480 grayscale ultrasound images to extract a binary mask, marking tumor voxels as 1 and background as 0. A postprocessing algorithm then computes the voxel-wise horizontal distance from the tumor centroid to the ultrasound frame’s vertical centerline.

To achieve low-latency inference with minimal resources, we optimized the model for the NVIDIA Jetson hardware. We used TensorRT [28] to recompile the original model (standard.onnx format) into an optimized file that takes into account the specific hardware features of the inference device. The inference application was built with the Holoscan SDK [29], which specializes in performing efficient inference with TensorRT models. For image acquisition, we used a Clarius C3HD probe [30], streaming raw video directly to the inference device via the Clarius Cast API [31].

### Experimental setup

**Fig. 6:**
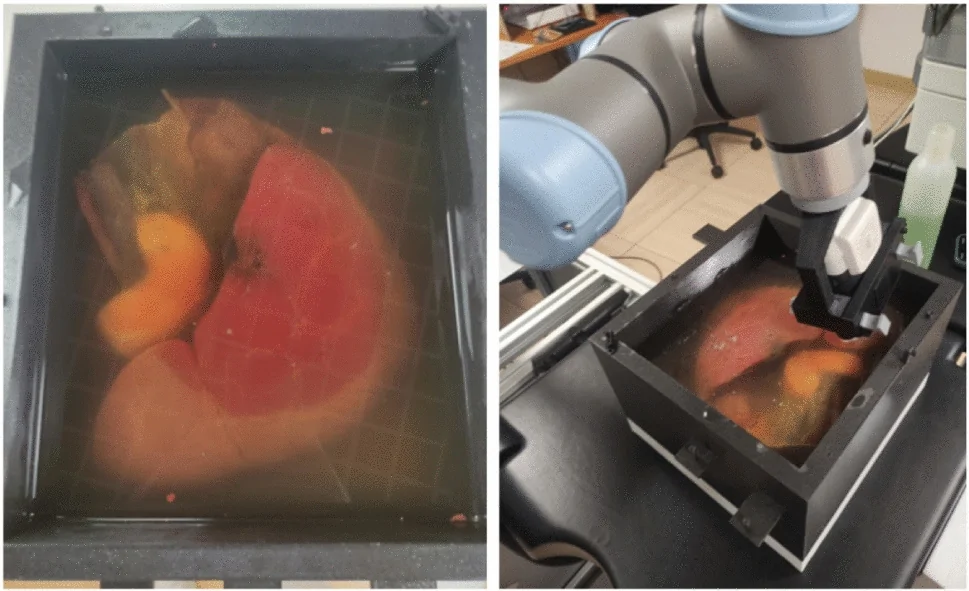
Left: Close up look of the validation phantom. Right: The robot scanning over the phantom

We tested the platform’s functionality for the use case of hepatic tumor ablation, where the ultrasound is used to localize solid tumors in the liver before needle insertion. To that purpose, we used a commercial liver phantom containing embedded tumor masses. To simulate skin and adipose tissue, we encased the phantom in a solid gelatin solution formulated to emulate real human tissue [32]. Figure 6 illustrates the validation setup.

#### Quantitative evaluation

Three trials were conducted:

- **Tumor localization: **We computed the average success rate and execution time of autonomous tumor localization tasks, by placing the probe in different initialization positions, distributed evenly over the phantom surface, and measuring in how many of the trials it was able to find and focus the tumor properly.
- **Tumor segmentation:** The Dice score and Intersection Over Union (IoU) were computed to assess the segmentation quality with respect to human-annotated ground truth masks in the 186 frames out of the 419 of the validation dataset that included tumoral tissue. We also computed the average segmentation time per frame.
- **Voice commands:** To validate the voice command interface, we calculated the system’s command identification success rate and its response latency.

#### Qualitative evaluation

To gather diverse perspectives, we demonstrated the platform to a panel composed by three experts of different backgrounds: one experienced surgeon, one founder of a surgical navigation equipment manufacturing company and a research center director with a broad experience in the fields of computer vision and medical image analysis, among others.

## Results

### Quantitative evaluation

Table 1 presents the obtained results’ summary.

**Table 1:** Quantitative result summary. The reported numbers are the average for the considered sample size, and the ± term corresponds to the standard deviation

**Tumor localization**	
Sample num (N)	25
Success rate	84 %
Execution time	5.75±7.13 s
**Tumor segmentation**	
Sample num (N)	186
Dice score	0.92±0.12
IoU	0.87±0.13
Execution time	0.1017 s
**Voice commands**	
Sample num (N)	100
Success rate	90 %
Response latency	2.69±0.78 s

We categorized failures in tumor localization as either settling on a false positive (no tumor) or failing to detect the tumor within a 60-second sweep. A high standard deviation in localization time reflects three specific cases that exceeded 10 s; the longest successful trial took 32 s.

### Qualitative evaluation: expert feedback

The experts remarked the intuitive control provided by the voice interface, the simple virtual scene and the augmented reality visualization. One key concept that attracted especial interest was the ability of the system to work in real-time, dynamically updating the position of the probe. Potentially, the system could use this capacity to adapt to patient breathing and motion. This feature cannot be exploited to its fullest in the current scenario, where tumor localization takes a short time and needle insertion needs to happen with no movement from the probe. However, they commented that with few changes the system could be extrapolated to other scenarios where surgeries take hours and accurate real-time ultrasound feedback is desirable but too tiring to be executed by a human operator. In this context, a robotic solution may be cost effective.

## Discussion

This paper presents an integrated robotic platform for manual and autonomous surgical ultrasound scanning and tumor localization. Its core strengths lie in its simple control algorithms, intuitive human–machine interface, and optimized AI infrastructure for fast inference in constrained environments.

The platform’s primary value stems from integrating diverse technological components into a unified system. We designed this platform to address real-world clinical restrictions, prioritizing user-friendly interfaces and hardware constraints. Rather than presenting an isolated AI model or control mode, we provide an end-to-end system for ultrasound navigation. Inspired by the “Three Computer Solution” for physical AI and robotics [14], our platform leverages three specialized computers–for training, control, and real-time inference–that can work in tandem to improve performance using new real-world data.

Regarding autonomy, we proposed a straightforward control mode: once the system detects a tumor, the robot follows predictable geometric rules to center it. Users monitor detections in real-time, identifying the robot’s trajectory in advance. If a surgeon identifies a potential issue or prefers manual adjustment, they can halt the robot and switch to manual mode using natural voice commands. Voice control is helpful for surgeons because it frees them from button- or screen-based interfaces when their hands are busy handling instruments.

In practice, our current reliance on cloud-based methods (Gemini, Whisper) for the voice command interface introduces a limitation, as cloud communication can be unreliable and fail to meet safety requirements. While this work presents a proof-of-concept scenario demonstrating that LLM-driven voice interaction is both technologically feasible and desirable, future implementations will host these models locally to eliminate connectivity issues.

Our phantom surface presents another limitation: we have only tested the robotic control module on planar surfaces of known length. Although the vertical force-control mode keeps the robot in constant contact with curved patient surfaces, image quality diminishes near highly curved borders. Furthermore, without a properly calibrated sweeping dimension, the arm could fall off and require relocation. Future path-planning strategies based on the patient’s geometry–extracted from the 3D Digital Twin–will help us tackle this issue.

Regarding the selected application, surgical robots are very expensive and difficult to bring to market, so it is critical to define accurately their specific aim and use case from the early stage of development, making sure they are cost effective. It is unfeasible to commercialize robots that perform tasks that require little time and effort, or robots that specialize in complex surgeries that rarely occur. We believe autonomous robots add the most value when performing simple but labor-intensive tasks over long durations. It is also important that the said tasks are performed frequently in regular clinical practice.

Ultrasound scanning fits this description. While surgeons recognize its value, clinical practice requires systems that adapt to real-time changes like breathing and motion–a significant challenge in soft-tissue surgery, because of the constant deformation. As demonstrated by this work, utilizing optimized AI tools (Holoscan SDK, TensorRT) and specialized edge devices (Jetson AGX Orin) enables systems that can adapt swiftly to sudden changes and operate autonomously through extended timespans. The latencies achieved here could be matched using a PC with enough GPU power, but the platform’s inference PC is not a standard GPU-equipped workstation, but an energy-efficient edge computer optimized to work in constrained scenarios, which brings our platform closer to a real clinical setting.

The platform’s modular architecture allows for relatively easy adaptation to other specialties. For example, to transition from liver tumors to other anatomical parts, we only need to replace the target segmentation model within the image analysis module. Similarly, users can add or remove visualization modes, such as the AR interface or the Digital Twin, by simply coupling or decoupling them to/from the existing system network. In future works, following clinician feedback to maximize the added value of the platform, we will aim to bring it to urology and neurosurgery, where procedures are longer and more effort-demanding.

## Conclusion

We present an integrated surgical robotic platform for ultrasound scanning based on: real-time image analysis for ultrasound target segmentation, robotic control based on visual servoing to locate and focus on the ultrasound target and a multimodal human–machine interface that allows the user to visualize the procedure on either a virtual scene or an AR interface and interact with the robot using natural voice commands. The platform has been designed to perform the scanning on either manual (cobot) or autonomous (visual servoing) mode.

The platform’s primary strength lies in its industrial-standard integration of diverse technologies and an optimized hardware infrastructure that ensures real-time performance within the constraints of an operating room.

We tested the platform on hepatic tumor localization using a synthetic liver phantom. Moving forward, we aim to adapt this technology for neurosurgical and urological procedures, where surgeons can fully leverage autonomous operation during long, demanding surgeries.
